# New Secondary Metabolites of Mangrove-Associated Strains

**DOI:** 10.3390/md22080372

**Published:** 2024-08-16

**Authors:** Yunxia Yu, Zimin Wang, Dingmi Xiong, Liman Zhou, Fandong Kong, Qi Wang

**Affiliations:** 1 Department of Pediatric Intensive Care Medicine, Hainan Women and Children’s Medical Center, Haikou 570206, China; yuyunxia2023@163.com; 2 Key Laboratory of Chemistry and Engineering of Forest Products, State Ethnic Affairs Commission, Guangxi Key Laboratory of Chemistry and Engineering of Forest Products, Guangxi Collaborative Innovation Center for Chemistry and Engineering of Forest Products, Guangxi Minzu University, Nanning 530006, China; wzm55802023@163.com (Z.W.); xdm7026@163.com (D.X.); zhouliman88@126.com (L.Z.)

**Keywords:** mangrove ecosystems, microorganisms, secondary metabolites, new compounds

## Abstract

Positioned at the dynamic interface between terrestrial and marine realms, mangroves embody a vibrant tapestry of biodiversity, encompassing an array of plants, animals, and microorganisms. These microbial inhabitants of mangrove habitats have emerged as a pivotal resource for antimicrobials and a plethora of pharmaceutically valuable compounds, spanning enzymes, antineoplastic agents, pesticides, immunosuppressants, and immunomodulators. This review delves into the recent landscape (January 2021 to May 2024, according to the time of publication) of novel secondary metabolites isolated from mangrove-associated microorganisms, analyzing 41 microbial strains that collectively yielded 165 distinct compounds. Our objective is to assess the productivity and potential of natural products derived from microbial populations within mangrove ecosystems in recent times. Notably, fungi stand out as the preeminent contributors to the emergence of these novel natural products, underscoring their pivotal role in the bioprospecting endeavors within these unique environments.

## 1. Introduction

Despite their fragility and sporadic distribution, mangrove ecosystems worldwide exhibit remarkable productivity [1]. These unique environments are marked by periodic tidal inundation, leading to significant fluctuations in environmental factors such as salinity and nutrient availability, thereby imparting specific and distinguishing traits [2]. The abundance of carbon and other essential nutrients within these ecosystems fosters the proliferation of diverse microbial communities, which have evolved remarkable resilience to moderate salinity levels and the unpredictable nature of their surroundings [3]. This microbial diversity encompasses a broad spectrum of organisms, including bacteria, fungi, cyanobacteria, microalgae, macroalgae, and fungus-like protists, all of which have been documented within mangrove habitats [4].

Natural products (NPs), encompassing the secondary metabolites extracted from animals, plants, marine organisms, and microorganisms, have garnered immense attention since the discovery of their unique physiological activities. This groundbreaking revelation has paved the way for the development of numerous therapeutic and healthcare drugs [5]. Notably, some of these natural products serve as lead compounds, undergoing strategic structural modifications to emerge as novel generations of drugs [6]. Furthermore, they boast significant economic value, finding applications in pesticides, food additives, daily chemicals, and other fine chemical products.

The exploration of bioactive lead compounds from mangrove microorganisms has emerged as a vibrant frontier within natural product chemistry research [7]. Mangrove-derived microbes represent a promising reservoir of bioactive natural products, yielding structurally unparalleled compounds with therapeutic potential [8,9]. However, the challenge remains for drug developers to effectively harness this abundant source of natural products. In recent years, particularly after 2013, Blunt and his colleagues have underscored the distinction between mangrove-associated fungi and marine fungi, driven by the proliferation of reported compounds originating from fungi inhabiting mangrove plants and soils [10,11,12,13]. This distinction underscores the uniqueness and potential of mangrove-derived microorganisms.

Recently, many researchers have reviewed mangrove-associated natural products from different aspects. Chen et al. summarized the discovery relating to the source and characteristics of metabolic products isolated from mangrove-associated fungi from 1989 to 2020, focusing on bioactivity and the unique chemical diversity of these natural products [3]. Braga et al. reviewed antibacterial, antifungal, and antiviral chemicals produced by soil/sediment-derived mangrove fungi from 1990 to 2022 [14]. Wu et al. collected 134 secondary metabolites and classified them into two major families in terms of the biological sources and 15 subfamilies according to the chemical structures, highlighting the structural diversity and bioactivities of the mangrove ecosystem-associated secondary metabolites [15]. Law et al. highlighted research on mangrove-derived streptomycetes and the production of anticancer-related compounds from these microorganisms (2008–2019) [16]. Collectively, these reviews underscore the immense untapped potential of mangrove microbial secondary metabolites as a rich source for the development of novel therapeutic agents in the medical realm. In this comprehensive review, we delve into the diverse strains sourced from mangroves, the myriad of compounds they produce, and the biological activities associated with these compounds. By examining the latest advancements and trends in this field, we aim to provide insights that may inspire future research endeavors and facilitate the discovery of novel bioactive natural products with significant therapeutic and economic implications.

## 2. Strains

**Figure 1:**
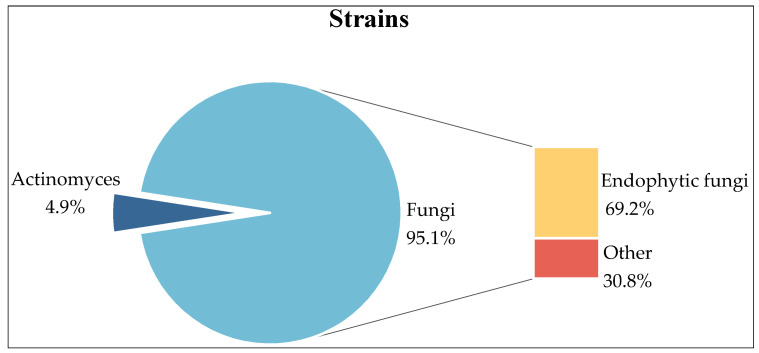
The types of strains.

Herein, 41 strains were reviewed, including 39 strains of fungi and 2 strains of actinomycetes, of which 27 strains of endophytic fungi accounted for 69.2% of the total fungi (Figure 1).

### 2.1. Fungi

**Figure 2:**
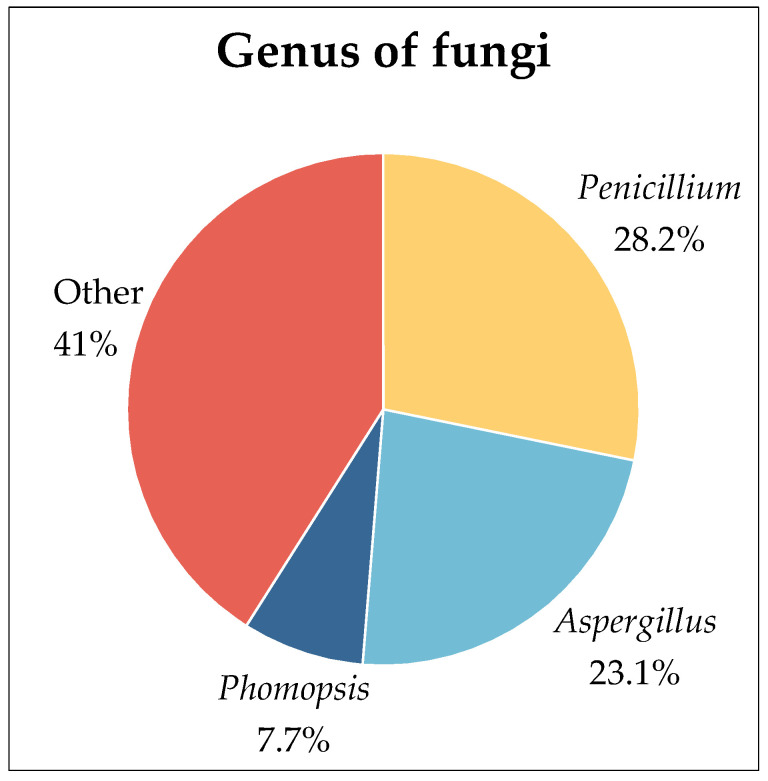
Genus of 39 strains of fungi.

Mangrove forests are biodiversity ‘hotspots’ for marine fungi [17]. Fungi are essential to the survival of this ecosystem. They participate in the synthesis of enzymes required for the decomposition of organic matter in this environment, converting it into nutrients available for its metabolism or that of other organisms, as well as allowing subsequent colonization by bacteria and yeasts to supplement the decomposition process, thereby contributing to the cycling and flow of nutrients to higher trophic levels [14,18,19,20]. The mangrove environment is an important target for bioprospection of secondary metabolite-producing fungi because it contributes to the development of several fungal species with potential biotechnological applications. After all, fungal secondary metabolites are typically produced in response to biotic or abiotic environmental influences. The organisms present in these areas are expected to be sources of unusual compounds due to the mangrove’s unique characteristics [21,22]. Currently, research on the secondary metabolites of fungi associated with mangroves has grown significantly. In this paper, the secondary metabolites of 39 strains of fungi were reviewed. Among these fungi, there were 11 strains of *Penicillium*, 9 strains of *Aspergillus*, and 3 strains of *Phomopsis*. There are 17 genera in total (Figure 2).

### 2.2. Actinomycetes

Actinomycetes are a potential source of bioactive substances and the most abundant source of secondary metabolites. Several reports from different geographical locations around the world have described the occurrences of actinomycetes in different mangrove habitats [23,24]. This relatively large distribution of actinomycete species in mangrove ecosystems worldwide seems to indicate that mangroves are a treasure trove of actinomycetes.

### 2.3. Endophytic Fungi

**Figure 3:**
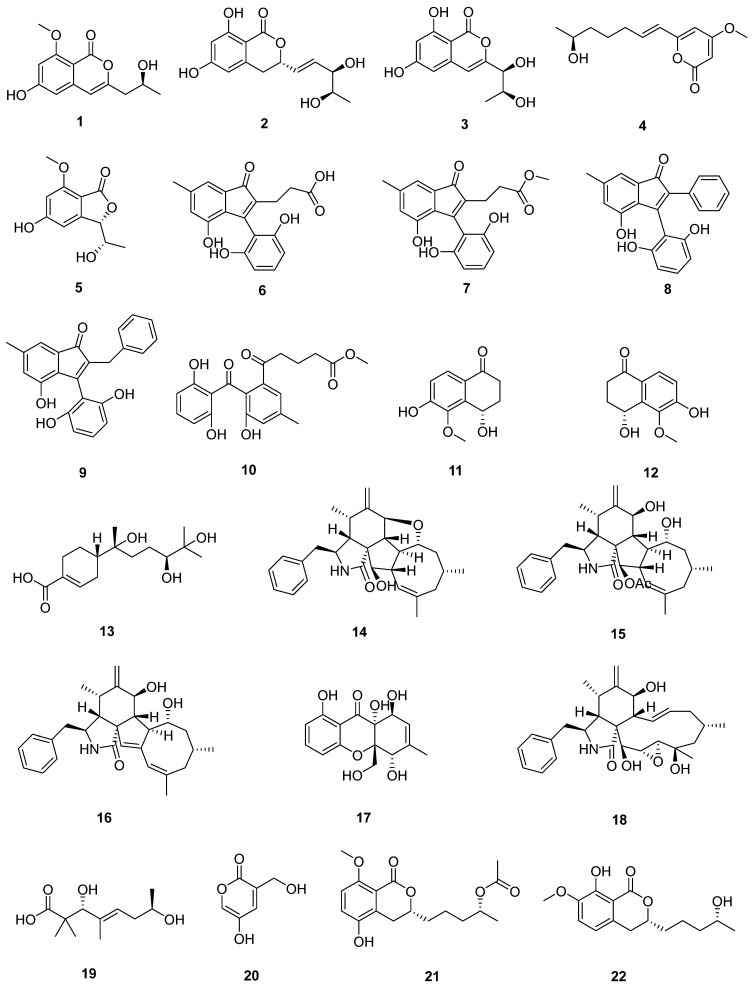

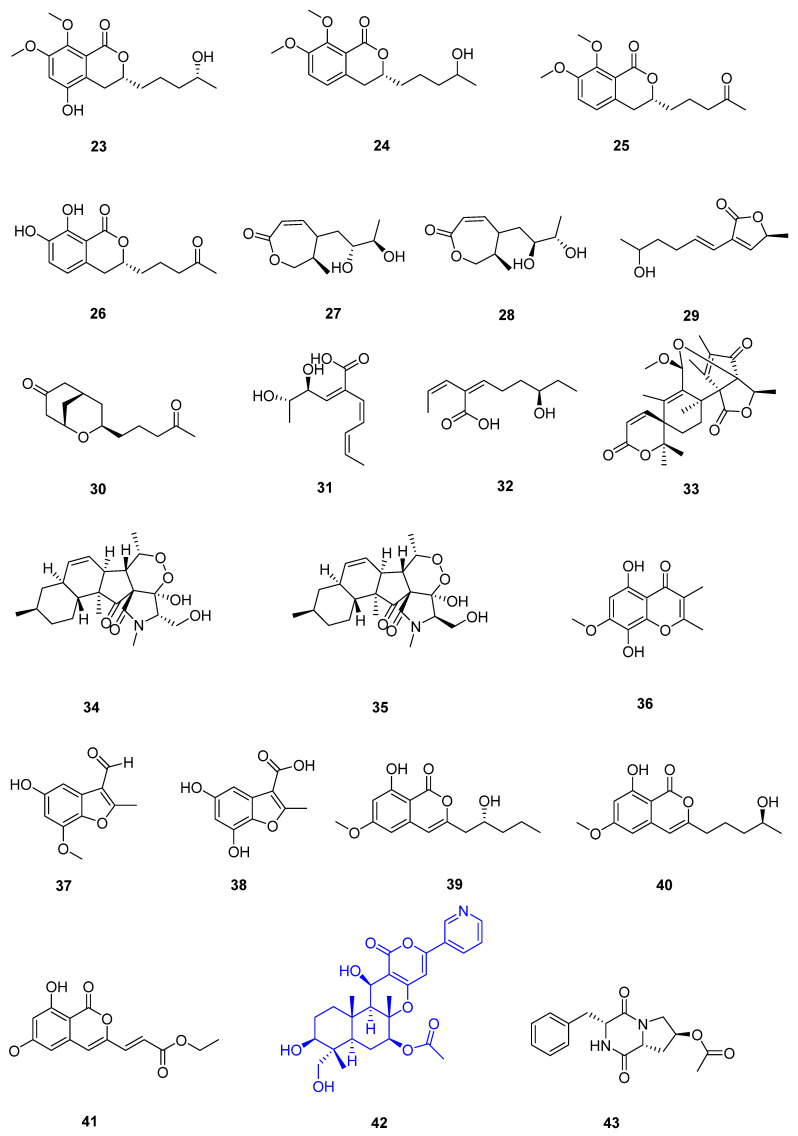

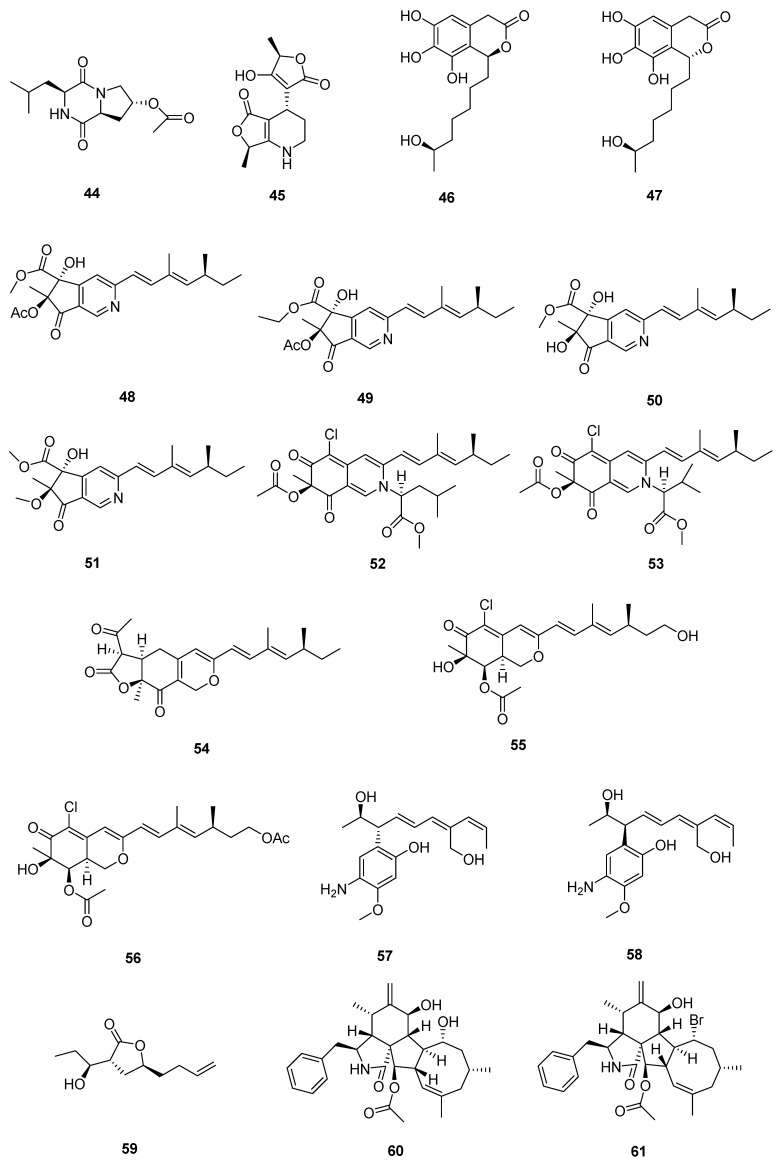

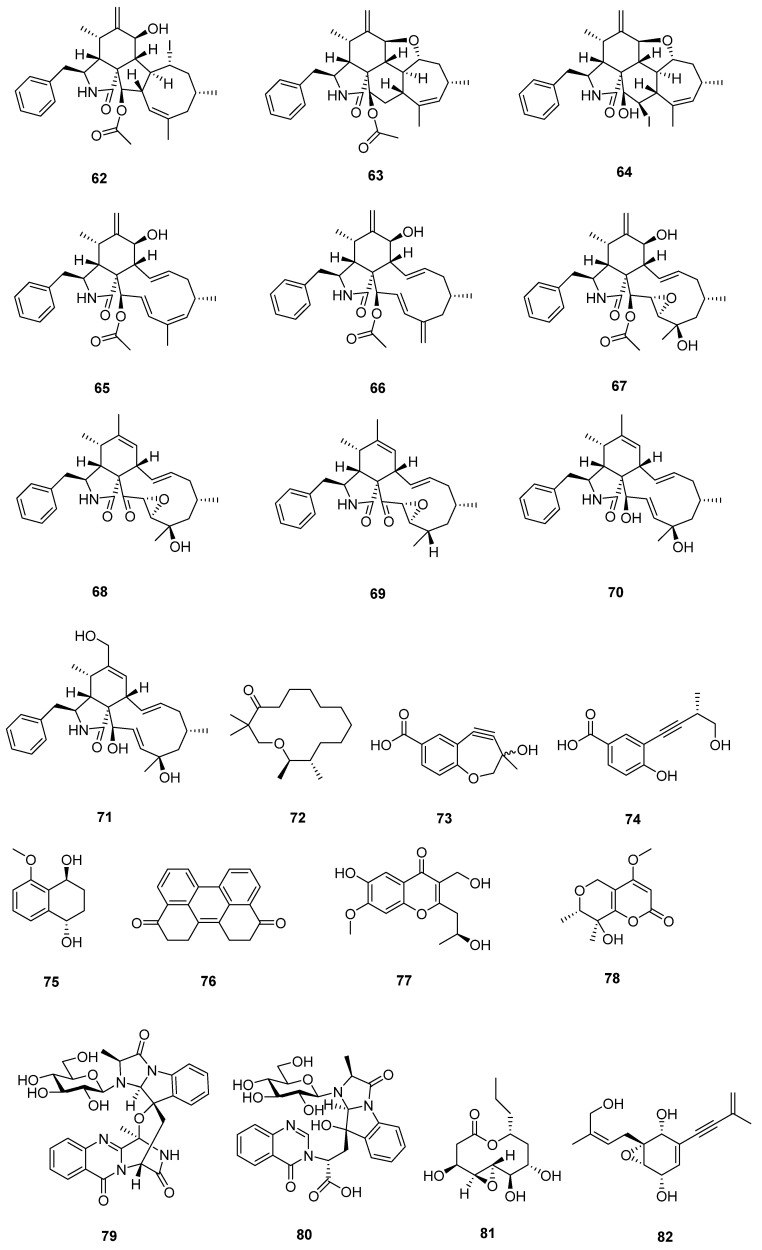

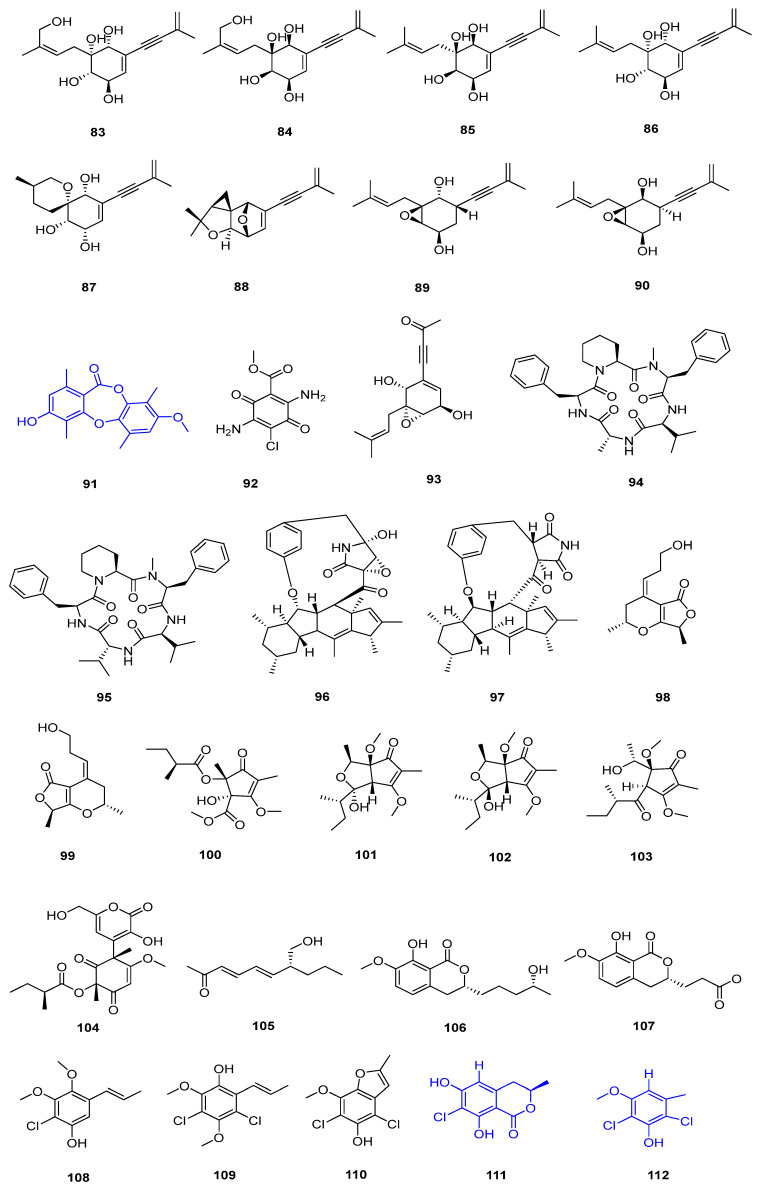
Compounds isolated from endophytic fungi. Note: new compounds are marked in black; the blue ones are new natural products.

**Figure 4:**
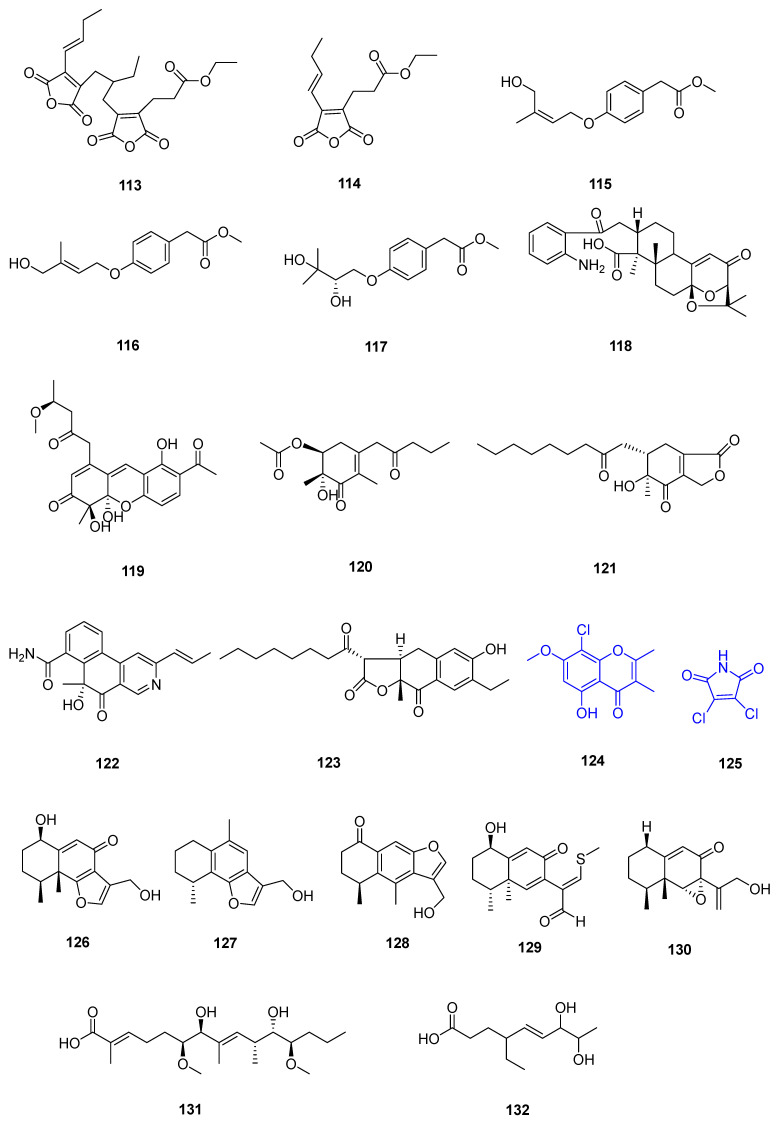

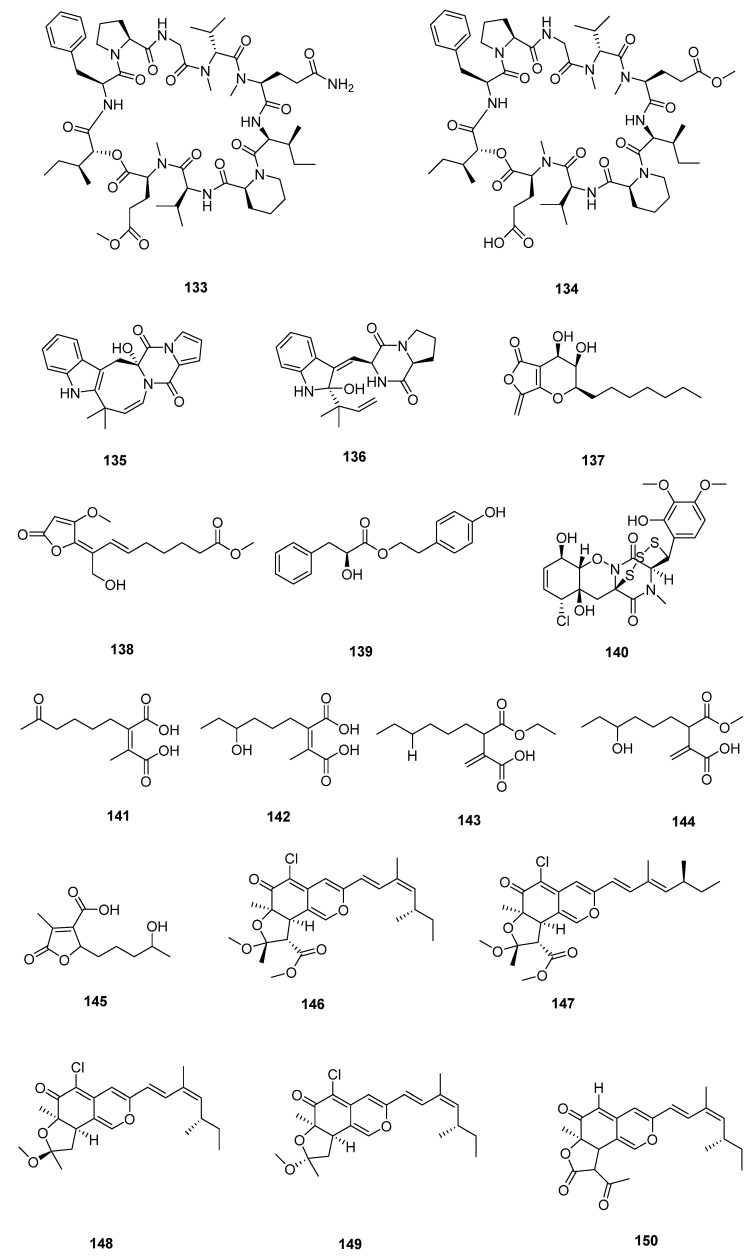

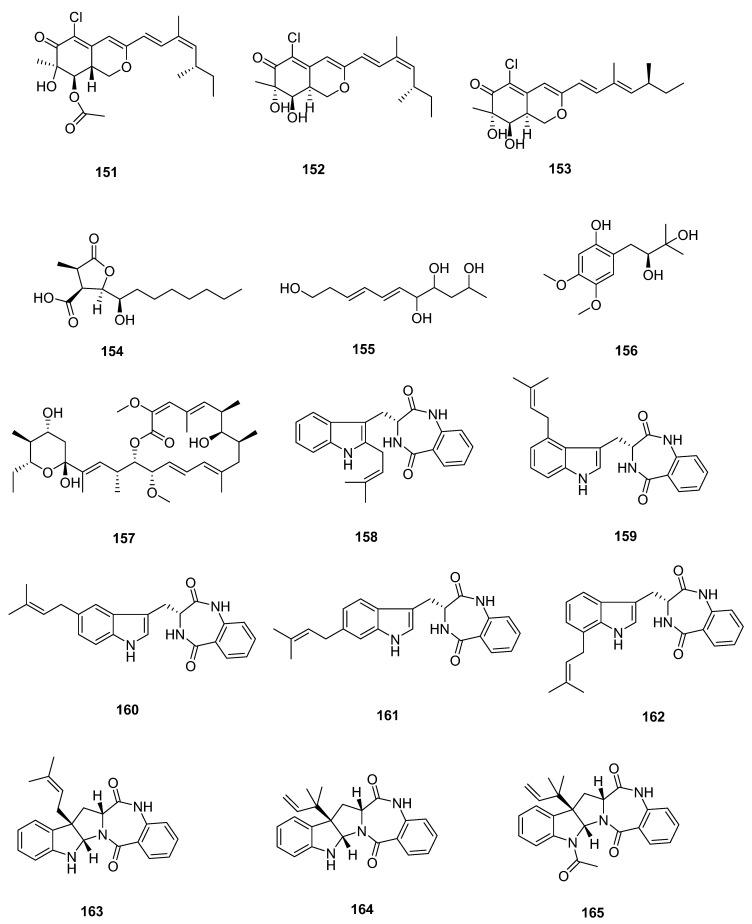
Compounds from other sources. Note: new compounds are marked in black; the blue ones are new natural products.

Endophytic fungi refer to fungi that live within plant tissues and spend part of their life cycle in plant systems without causing any obvious pathogenic symptoms [25]. Most endophytic fungi belong to the genus Ascomycetes and are a multi-class group [3]. Endophytic fungi are an important class of mangrove fungi, and mangrove endophytic fungi are the second largest group of marine fungi, and the study of secondary metabolites is a promising new field [26,27]. Mangrove endophytic fungi have evolved in symbiosis with host plants, forming a unique bioactive substance synthesis pathway or metabolic pathway, from which a wealth of novel structures and/or bioactive substances with special functions can be metabolized, such as antitumor, antibiotic, neuroprotective, antioxidant, anti-inflammatory, antiviral and immunomodulator compounds [3,25]. Herein, 112 secondary metabolites isolated from 27 strains of endophytic fungi and their biological activities were reviewed (Table 1) (Figure 3 and Figure 4).

## 3. Compounds

**Figure 5:**
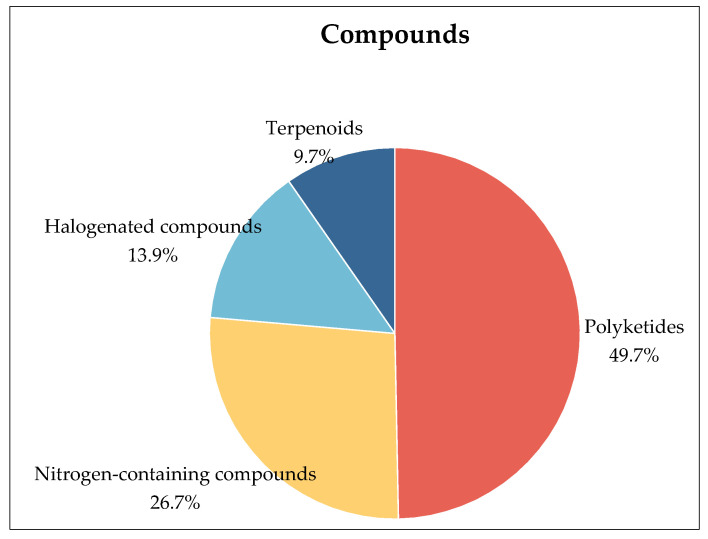
Types of all compounds.

Among the 165 new natural products, there are mainly polyketides, nitrogen-containing compounds, halogenated compounds, and terpenoids, including a pair of tetralone enantiomers, a pair of mutually converting epimers and a pair of new enantiomers (+) and (−) didymetone (among these compounds, some nitrogen-containing and halogenated compounds are also polyketides) (Figure 5).

### 3.1. Polyketides

Polyketides represent a highly diverse group of natural products with structurally intriguing carbon skeletons, which comprise polyphenols, macrolides, polyenes, ene-diynes, and polyethers [58].

Three new isocoumarins (**1**–**3**) and one new pyrone derivative (**4**) were isolated from the ethyl acetate extract of the fermentation broth of the mangrove endophytic fungus *Phomopsis* sp. DHS-11 [28]. And a novel benzofuranone compound (**5**) was discovered for the first time from its fermented extract [29]. Seven new polyketides, including four indenone derivatives, cytoindenones A–C (**6**, **8**–**9**), 3′-methoxycytoindenone A (**7**), a benzophenone derivative, cytorhizophin J (**10**), and a pair of tetralone enantiomers, (±)-4,6-dihydroxy-5-methoxy-α-tetralone (**11** and **12**) were obtained from the endophytic fungus *Cytospora heveae* NSHSJ-2 isolated from the fresh stem of the mangrove plant *Sonneratia caseolaris* [30]. A mangrove endophytic fungus *Phomopsis asparagi* DHS-48 was found to be particularly productive, and one new compound named phaseolorin J (**17**) was isolated from the culture treated with sodium butyrate [32,33]. Two new polyketides, pestalotiopin B (**19**) and pestalotiopyrone N (**10**) were obtained from the ethyl acetate extracts of the rice solid cultures of the mangrove endophytic fungus *Pestalotiopsis* sp. HQD-6 [34]. Six new isocoumarin derivatives talaromarins A–F (**21**–**26**) were isolated from the mangrove-derived fungus *Talaromyces flavus* TGGP35 [35]. Six new polyketides, which included three new lactones (talarotones A–C) (**27**–**29**), one new polyketide (talarotide A) (**30**), and two new polyenes (talaroyenes A and B) (**31**, **32**), were isolated from the mangrove-derived fungus *Talaromyces flavus* TGGP35 [36]. One new chromone fusarimone A (**36**), two new benzofurans fusarifurans A (**37**) and B (**38**), and three new isocoumarins fusarimarins A–C (**39**–**41**) were isolated from the mangrove endophytic fungus *Fusarium* sp. 2ST2 [37]. Two new octaketides, cytosporones W (**46**) and X (**47**), were isolated from the mangrove endophytic fungus *Diaporthe* sp. ZJHJYZ-1. Compounds **46** and **47** were a pair of epimers, whose configuration of C-1 could mutually convert, causing racemization of the lactone ring [41]. One undescribed azaphilone derivative (**54**) was obtained and identified from the fermented rice cultures of a mangrove endophytic fungus *Penicillium sclerotiorum* ZJHJJ-18 [42]. One new long-chain polyene pinophol G (**59**) was obtained from EtOAc extract of the mangrove-derived fungus *Penicillium herquei* JX4 [43]. Five new polyketide derivatives, eschscholin B (**72**), dalditone A and B (**73** and **74**), (1R,4R)-5-methoxy-1,2,3,4-tetrahydronaphthalene-1,4-dio (**75**), and daldilene A (**76**), were isolated from the mangrove endophytic fungus *Daldinia eschscholtzii* KBJYZ-1 [45]. Two new pyrone derivatives, 2-(12S-hydroxypropyl)-3-hydroxy-methyl-6-hydroxy-7-methoxychromone (**77**) and (±)-pyrenocine S (**78**), were obtained from the mangrove endophytic fungus *Aspergillus sydowii* #2B [46]. A new lactone, asperlactone A (**81**), was isolated from the mangrove endophytic fungus *Aspergillus* sp. GXNU-A9 [48]. The mangrove endophytic fungus *Aspergillus* sp. GXIMD00016 was fermented by using rice medium. The metabolites were isolated by chromatography technology and 2,7-didechlorovicanic (**91**) were obtained [50]. A new diisoprenyl-cyclohexene-type meroterpenoid, biscognienyne M (**93**), was isolated from the mangrove endophytic fungus *Aspergillus* QG1a [51]. A pair of new enantiomers (+) and (−) didymetone (**98** and **99**) were purified from the mangrove endophytic fungus *Didymella* sp. CYSK-4 [53]. Five undescribed polyketides, including two talaketide derivatives (**100** and **101**), two asperpentenone derivatives (**102** and **103**), and one phomaligol derivative (**104**), were obtained from the mangrove endophytic fungus *Fusarium proliferatum* NSD-1 [54]. A new compound, named penicillquei C (**105**), was isolated from the fermentation broth of the mangrove-derived fungus *Penicillium verruculosum* TGM14 [55]. Two new isocoumarins named peniciisocoumarins I and J (**106** and **107**) were obtained from *Penicillium* sp. GXIMD 03001, an endophytic fungus derived from the rhizophoraceous mangrove *Kandelia candel* [56].

To discover bioactive natural products from mangrove sediment-derived microbes, a chemical investigation of the two Beibu Gulf-derived fungal strains, *Talaromyces* sp. SCSIO 41050 and *Penicillium* sp. SCSIO 41411, led to the isolation of 23 natural products. Five of them were identified as new ones, including two polyketide derivatives with unusual acid anhydride moieties named cordyanhydride A ethyl ester (**113**) and maleicanhydridane (**114**), and three hydroxyphenylacetic acid derivatives named stachylines H–J (**115**–**117**) [59]. Through the thorough investigation into the chemical constituents of *M. purpureus* wmd2424, five previously undescribed compounds, monascuspurins A–E (**119**–**123**), were isolated from the EtOAc extract of the mangrove-derived fungus *Monascus purpureus* wmd2424 cultured in RGY medium [60]. Two previously undescribed linear polyketides **131**–**132** were identified by spectroscopic methods from the culture broth of the mangrove-derived actinomycete *Streptomyces* sp. WHUA03072 [61]. Three new polyketides penicinones A–C (**137**–**139**) were isolated and identified from the culture extract of the mangrove-derived fungus *Penicillium* sp [62]. Five new alkane derivatives (**141**–**145**) were isolated from the mangrove sediment-derived fungus *Penicillium ludwigii* SCSIO 41408 [63]. Ochlephilone **150** was isolated from the culture broth of the mangrove-derived fungus *Penicillium sclerotiorum* HY5 [64]. Chemical investigation of the fungus *Xylariaceae* sp. SCSIO41212 has led to the isolation of two new compounds, xylaolide B (**154**) and xylaolide C (**155**) [65]. A novel tetrasubstituted benzene derivative peniprenylphenol A (**156**) was isolated from a scaled-up culture of the Indonesian mangrove sediment-derived fungus *Penicillium chrysogenum* ZZ1151 in rice medium [66]. An active compound **157** was isolated from the fermentation broth of *Streptomyces* sp. MCCG218 [67].

### 3.2. Nitrogen-Containing Compounds

Nitrogen-containing compounds are core parts not only of natural and synthetic medicines but also of biologically active compounds including natural products [68].

Three new cytochalasins, phomoparagins A–C (**14**–**16**), were isolated from *Phomopsis asparagi* DHS-48, a mangrove-derived endophytic fungus [32,33]. Compound phomoparagin D (**18**) was isolated from the culture treated with sodium butyrate of the fungus [32,33]. Two new 3-decalinoyltetramic acid derivatives with peroxide bridge fusarisetins E (**34**) and F (**35**) were isolated from the mangrove endophytic fungus *Fusarium* sp. 2ST2 [37]. Chemical investigation of endophytic fungus *Aspergillus fumigatus* HQD24, isolated from the flower of *Rhizophora mucronata*, led to the isolation of eight alkaloids, in which compound **42** was known as a synthetic product and isolated as a natural product for the first time [38]. Two new 2,5-diketopiperazines derivatives (**43**–**44**) were isolated from a culture broth of an endophytic fungus *Nigrospora camelliae-sinensis* S30, derived from mangrove *Lumnitzera littorea* [39]. One previously undescribed alkaloid, named penifuranone A (**45**), was isolated from the mangrove endophytic fungus *Penicillium crustosum* SCNU-F0006 [40]. Four undescribed azaphilone derivatives, sclerazaphilones A–D (**48**–**51**) were obtained and identified from the fermented rice cultures of a mangrove endophytic fungus *Penicillium sclerotiorum* ZJHJJ-18 [42]. One new epimer pair of long-chain polyenes penicilqueis E (**57**) and F (**58**) was obtained from EtOAc extract of the mangrove-derived fungus *Penicillium herquei* JX4 [43]. Nine new cytochalasins (**60**, **63**, **65**–**71**) were isolated from the mangrove-derived fungus *Phomopsis* sp. QYM-13 [44]. Two new glucosidated indole-containing quinazoline alkaloids designated fumigatosides G (**79**) and H (**80**) were isolated from the mangrove-derived fungus *Aspergillus fumigatus* SAl12 [47]. An investigation on bioactive metabolites from the mangrove endophytic fungus *Aspergillus* sp. GXNU-4QQY1a led to the isolation of two undescribed cyclic peptides, guaspertide A (**94**) and guaspertide B (**95**) [52]. Two new 12- or 13-membered-ring macrocyclic alkaloids ascomylactam D and E (**96**, **97**) were purified from the mangrove endophytic fungus *Didymella* sp. CYSK-4 [53].

An unprecedented di-*seco*-indole diterpenoid, peniditerpenoid A (**118**), was obtained from the mangrove-sediment-derived fungus *Penicillium* sp. SCSIO 41411 [69]. Chemical examination of the fermented broth of the mangrove-derived fungus *Phaeosphaeriopsis* sp. S296 resulted in the isolation of two new cyclodecadepsipeptides, namely phaeosphamides A (**133**) and B (**134**) [70]. Two new prenylated indole diketopiperazine alkaloids (PIDAs) penicamides A and B (**135** and **136**) were isolated and identified from the culture extract of the mangrove-derived fungus *Penicillium* sp. [62]. Four new alkaloid compounds asperkaloids A–D (**158**–**161**) and four new indole-benzodiazepine-2,5-dione derivatives asperdinones E–H (**162**–**165**) were isolated from the culture extracts of the mangrove-derived fungus *Aspergillus spinosus* WHUF0344 [71].

### 3.3. Terpenoids

Terpenoids are the most abundant class of compounds in natural substances. There are many terpenoids with strong physiological or biological activity that have been used in clinical practice, and the well-known anti-malarial drug artemisinin and the antitumor drug paclitaxel belong to this family. At the same time, they are also an important class of natural flavors, which are indispensable raw materials for the cosmetics and food industries.

A new polychiral bisabolane sesquiterpene, bisabolanoic acid A (**13**), was isolated from the mangrove-derived fungus *Colletotrichum* sp. SCSIO KcB3–2 [31]. One new meroterpenoid (talaropenoid A) (**33**) was isolated from the mangrove-derived fungus *Talaromyces flavus* TGGP35 [36]. Nine previously undescribed diisoprenyl-cyclohexene-type meroterpenoids, aspergienynes A–I (**82**–**90**), were obtained from the mangrove endophytic fungal strain *Aspergillus* sp. GXNU-Y65 [49].

Five new sesquiterpenoids, citreobenzofuran D–F (**126**–**128**) and phomenone A–B (**129**–**130**), were isolated from the culture of the mangrove-derived fungus *Penicillium* sp. HDN13-494 [72].

### 3.4. Halogenated Compounds

Marine halogenated compounds comprise a varied assembly of compounds, ranging from peptides, polyketides, indoles, terpenes, acetogenins, and phenols to volatile halogenated hydrocarbons [73]. Halogenation often provides these compounds with interesting key features [74].

Three new chlorinated compounds, including two propenylphenol derivatives, chlorophenol A and B (**108** and **109**), and one benzofuran derivative, chlorophenol C (**110**), were isolated from the mangrove endophytic fungus *Amorosia* sp. SCSIO 41026. 7-Chloro-3,4-dihydro-6,8-dihydroxy-3-methylisocoumarine (**111**) and 2,4-dichloro-3-hydroxy-5-methoxy-toluene (**112**) were obtained as new natural products [57]. Compounds **52**, **53**, **55**, and **56** were obtained and identified from the fermented rice cultures of a mangrove endophytic fungus *Penicillium sclerotiorum* ZJHJJ-18 [42]. One brominated (**61**) and two iodinated cytochalasins (**62** and **64**) were isolated from the mangrove-derived fungus *Phomopsis* sp. QYM-13 treated with 3% NaBr or 3% KI in potato liquid medium [45]. A new benzoquinone, guxiumasperone A (**92**), was isolated from the mangrove endophytic fungus *Aspergillus* QG1a [51].

Two new chlorinated metabolites, 8-chlorine-5-hydroxy-2,3-dimethyl-7-methoxychromone (**124**) and 3,4-dichloro-1H-pyrrole-2,5-dione (**125**), were isolated from the mangrove sediment-derived fungus *Mollisia* sp. SCSIO41409 [75]. A new trithiodiketopiperazine derivative, adametizine C (**140**), was isolated from the mangrove sediment-derived fungus *Penicillium ludwigii* SCSIO 41408 [63]. Chlorinated compounds **146**–**149** and **151**–**153** were isolated from the culture broth of the mangrove-derived fungus, *Penicillium sclerotiorum* HY5, by various chromatographic methods [64].

## 4. Bioactivity

**Figure 6:**
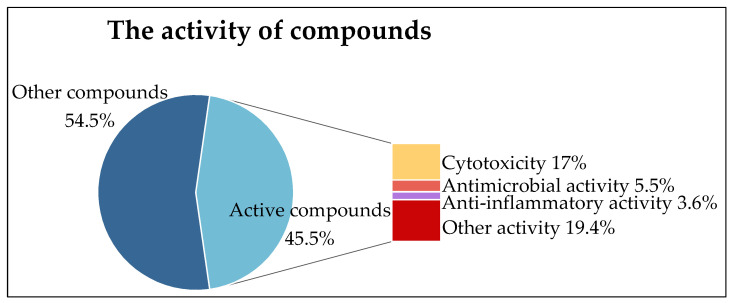
The activity of compounds.

Natural products have been relevant sources for drug discovery and the development of medicines since ancient times. Newman and Cragg showed that from January 1981 to September 2019, at least 1881 new drugs were approved worldwide for the treatment of all types of diseases, and among them, 75.38% (1418) were derived from natural products [76,77]. Among the 165 compounds mentioned in this paper, 75 compounds (45.5%) had biological activity, including cytotoxicity, antibacterial activity, anti-inflammatory activity, antioxidant activity, and other activities (active compounds here refer to compounds that are specifically active in the articles reviewed, and some of the weakly active compounds are not counted). Cytotoxic compounds accounted for 37.3% of the active compounds and 17% of the total compounds (Figure 6). It is worth mentioning that compounds **45**, **108**, **109**, **120**, **135**, and **138** have multiple activities at the same time [40,59,63,75].

The activity of endophytic fungal-derived compounds **1**–**112** is shown in Table 1. Then, the activity of other compounds (**113**–**165**) will be discussed next.

A variety of bioactive screens revealed polyketide derivative (**113**) to have obvious antifungal activity at 10 μmol L^−1^, exhibiting obvious inhibition against phosphodiesterase 4 (PDE4) with an inhibitory rate of 49.7%, and **114** displayed moderate cytotoxicity against cell lines A549 and WPMY-1. Compounds **115** and **116** showed potential to inhibit acetylcholinesterase (AChE) by an enzyme activity test, as well as in silico docking analysis [59]. Peniditerpenoid A (**118**) inhibited lipopolysaccharide-induced NF-κB with an IC_50_ value of 11 μmol L^−1^ and further effectively prevented RANKL-induced osteoclast differentiation in bone marrow macrophages [69]. Compounds (**121**–**123**) possessed mild antifungal activity against *Aspergillus niger*, *Penicillium italicum*, *Candida albicans*, and *Saccharomyces cerevisiae* [60]. Compound **125** showed antimicrobial activities against several pathogenic fungi and bacteria, and antiproliferative activities against two human prostate cancer cell lines (IC_50_ values 2.77 to 9.60 μmol L^−1^) [63]. Compound phomenone B (**130**) showed moderate activity against *Bacillus subtilis*, with an MIC value of 6.25 μmol L^−1^ [72]. Compound **133** showed inhibitory activities against tumor cell lines of AGS, BEL-7402, HepG2, B16, and BIU87 with IC_50_ values ranging from 5.14 to 66.38 μmol L^−1^ [70]. Compound **137** displayed potent cytotoxicity against murine melanoma (B16) cells, human breast adenocarcinoma (MCF-7) cells, and human hepatocellular carcinoma (HepG2) cells at 50.0 μmol L^−1^ with inhibitory ratios of 82.7%, 75.1%, and 95.9%, respectively [62]. In a variety of bioactivity screenings, compound **140** showed cytotoxicity against prostate cancer cell line 22Rv1. Adametizine C (**140**), with the strongest inhibitory activity against RANKL-induced osteoclast differentiation in bone marrow macrophage cells at 10 μmol L^−1^, was suggested to be the promising lead compound for the treatment of osteoclast-related diseases [63]. Compounds **149** and **150** exhibited potent phytotoxicity against the growth of radicle and plumule on *Amaranthus retroflexus* L., with EC_50_ values ranging from 234.87 to 320.84 μmol L^−1^ [64]. New peniprenylphenol A (**156**) was found to have antimicrobial activity against human pathogenic methicillin-resistant *Staphylococcus aureus* (MRSA), *Escherichia coli*, and *Candida albicans* with MIC values of 6, 13, and 13µg mL^−1^, respectively [66]. Compound **157** showed strong cell proliferation inhibitory activity against nasopharyngeal carcinoma cell lines TW03 and 5-8F, and the semi-inhibitory concentration (half-inhibitory concentration, IC_50_) was 2.7 μmol L^−1^ and 9.2 μmol L^−1^, respectively [67]. Compounds **162**, **163,** and **165** exhibited moderate inhibitory effects against α-glucosidase with IC_50_ values in the range of 24.65–312.25 μmol L^−1^ [71].

Among these compounds, compounds **50**–**52** exhibited stronger inhibitory effects on LPS-induced NO production in RAW264.7 macrophage cells than that of the positive control indomethacin and had no toxicity towards macrophage RAW 264.7 at 50 μmol L^−1^ [42]. This means azaphilone derivatives from mangrove fungi have a good chance of contributing to the discovery of potential anti-inflammatory agents. Compound **113** showed obvious antifungal activities, especially against *F. graminearum*, *F. oxysporum*, and *R. solani*, with MIC values of 6.25–12.5 µg mL^−1^, having the potential to be further developed into antifungal drugs [59]. Compounds **3**, **18**, **34**, **35**, **61**, **92**, **93**, **97**, **125**, and **137** have significant cytotoxic activity against selected tumor cells and have potential to be further developed into anticancer drugs [28,33,37,44,51,53,62,75], which deserve further attention. Compound **133** demonstrated highly selective inhibitory action towards AGS cells, specifically by arresting their cell cycle progression at the G2 phase and triggering apoptosis in a precise, dose-responsive manner [70]. This finding underscores the potential of Compound **133** as a promising lead candidate for the development of therapeutic strategies aimed at treating gastric adenocarcinoma.

### 4.1. Cytotoxicity

Cancer is the leading cause of death and an important barrier to increasing life expectancy around the world [78]. Currently, there is a great demand for new oncological therapies that reduce or do not cause severe adverse effects to patients, providing an improvement in quality of life. In this context, several studies seek new antitumor molecules from natural sources, attempting to reduce treatment costs, increase specificity, and decrease the side effects [79]. Various tumor cell lines from human and animal sources have been useful and applied for in vitro cytotoxicity and in vivo studies of the anticancer potential of natural products, whether by the academy or by pharmaceutical companies [76]. In this paper, 28 compounds showed cytotoxicity, accounting for 17% of the total compounds.

### 4.2. Antimicrobial Activity

Antimicrobial activity refers to the ability of a drug to inhibit or kill pathogenic microorganisms to treat infectious diseases. Antimicrobials include antibiotics, antifungals, and antivirals that kill microorganisms or inhibit their growth and reproduction by blocking key physiological functions of bacteria, fungi, or viruses. Compounds **45**, **114**–**118**, and **125** showed moderate antimicrobial activity [40,60].

### 4.3. Anti-Inflammatory Activity

Inflammation is an innate immunity that is the biological response of human tissues to various harmful stimuli. It is known to be a normal bodily defense mechanism that is activated in the event of injury, exposure to pollutants, radioactive materials, poisons, and allergens, as well as infection by various substances such as microorganisms, viruses, etc. Natural products play an important role in human health in the prevention and treatment of inflammation. Compounds **45**, **57**–**59**, **72**, **76**, and **81** showed anti-inflammatory activity [40,43,45,48].

### 4.4. Antioxidant Activity

Because of restriction on synthetic antioxidants due to their carcinogenicity, interest has increased considerably in finding naturally occurring antioxidants for use in foods, cosmetics, or medicine materials to replace the synthetic ones [80]. Among them, compounds **6**–**10**, **26**, and **31** had significant antioxidant activity [30,35,36].

## 5. Discussion

The microbial diversity within mangrove ecosystems is exceptionally rich and varied, harboring significant biotechnological potential. In a comprehensive review spanning 2021 to the present, we have examined the discovery of 165 novel secondary metabolites derived from 41 distinct microbial strains. Over the past four years, this research has unveiled 82 polyketides, 44 nitrogen-containing compounds, 16 terpenoids, and 23 halogenated compounds. Notably, some of the halogenated and nitrogen-containing compounds are also polyketides. Thus, polyketides dominate the metabolic landscape of mangrove-derived strains, while halogenated compounds also constitute a notable proportion. In terms of functional activity, nearly half of these secondary metabolites exhibit one or more biological activities, underscoring their potential as a foundation and roadmap for the discovery of novel drug leads.

However, our observation reveals that in recent years, there has been a notable surge in research endeavors focusing on the secondary metabolites of fungi, whereas a comparative scarcity of studies has emerged concerning the secondary metabolites of actinomycetes and bacteria. Given this disparity, it is prudent to redirect our attention and invest more efforts towards the exploration of actinomycetes and bacteria, as their secondary metabolites hold immense potential for scientific discovery and practical applications.

**Figure 7:**
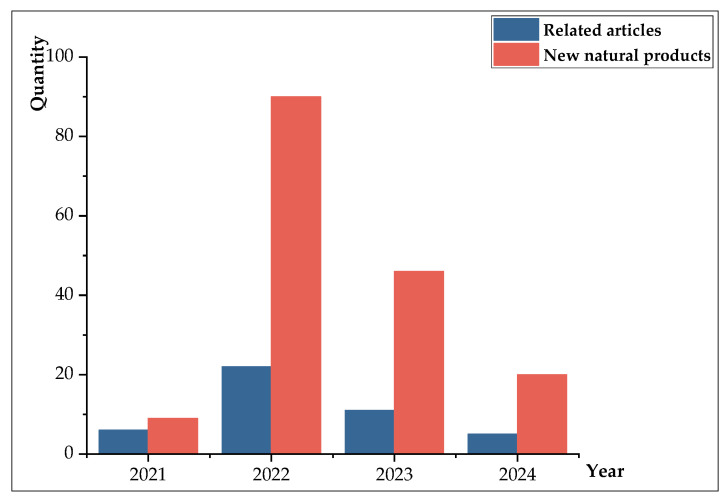
The number of relevant articles and new natural products in recent years.

Over the past four years, we have witnessed a notable trend in the discovery of new natural products. In 2021, six related articles reported a total of nine novel natural products. This number significantly increased in 2022, with 22 articles yielding an impressive 90 new natural products. In 2023, while still substantial, the number of articles declined to 11, documenting 46 new natural products. From January to May 2024, we observed five articles contributing 20 new natural products (exclusively counting articles dedicated to the discovery of novel natural products). This analysis highlights that 2022 witnessed the highest number of new natural products discovered, closely followed by 2023 (as depicted in Figure 7). One plausible explanation for this surge in 2022 could be attributed to the outbreak of the pandemic, which restricted human mobility and activities, potentially allowing researchers to dedicate more time and energy to their research endeavors, ultimately leading to a proliferation of publications.

With the swift advancements in synthetic biology technologies, including genome mining and enzyme catalysis, researchers are empowered with an arsenal of tools. These tools facilitate computational excavation of genetic data, enabling seamless correlations with known secondary metabolites, and a wealth of reviews document their utility and applications [81]. Furthermore, the incorporation of enzymes in natural product synthesis holds immense potential to revolutionize and streamline overall synthetic processes [82]. These innovative techniques can be seamlessly integrated into the exploration of mangrove microorganism-derived natural products, thereby accelerating the discovery of marine drugs originating from mangroves.

Natural products have long been instrumental in advancing human health and wellbeing, particularly in the realm of cancer prevention and treatment strategies. With advancements in laboratory technology and enhanced capabilities for strain isolation, researchers are increasingly empowered to extract and purify bioactive compounds. However, to fully harness the therapeutic potential of these compounds, it is imperative to deepen our understanding of their mechanisms of action within living organisms and cells. Furthermore, rigorous research is needed to validate the efficacy of these active compounds as potential novel drugs and lead structures for disease management, thereby fostering the development of innovative therapeutic interventions.

## Figures and Tables

**Table 1:** Secondary metabolites isolated from endophytic fungi and their activities.

Sr. No.	Endophytic Fungus	Host Plant(s)	Sources	Compound Isolated	Bioactivity and IC_50_/EC_50_/Inhibition	Reference
1	*Phomopsis* sp. DHS-11	the living root of the mangrove plant *Rhizophora mangle*	Dong Zhai Gang mangrove garden on Hainan Island, China	**1**	HeLa cells (11.49 ± 1.64 μmol L^−1^)	[28]
				**2**		
				**3**	HeLa cells (8.70 ± 0.94 μmol L^−1^)	
				**4**	HepG 2 (34.10 ± 2.92 μmol L^−1^)	
				**5**		[29]
2	*Cytospora heveae* NSHSJ-2	the fresh stem of mangrove plant *Sonneratia caseolaris*	the Nansha Mangrove National Nature Reserve in Guangdong Province, China	**6**	DPPH radical scavenging activity (11.5 ± 0.1 μmol L^−1^)	[30]
				**7**	DPPH radical scavenging activity (21.5 ± 1.0 μmol L^−1^)	
				**8**	DPPH radical scavenging activity (19.7 ± 1.8 μmol L^−1^)	
				**9**	DPPH radical scavenging activity (16.6 ± 0.4 μmol L^−1^)	
				**10**	DPPH radical scavenging activity (9.5 ± 0.1 μmol L^−1^)	
				**11**		
				**12**		
3	*Colletotrichum* sp. SCSIO KcB3-2	a mangrove plant, *Kandelia candel*	Dayawan, Shenzhen, Guangdong Province, China	**13**	AChE inhibitory activity (2.2 ± 0.18 μmol L^−1^)	[31]
4	*Phomopsis asparagi* DHS-48	a healthy tree root of the mangrove plant *Rhizophora mangle*	Dong Zhai Gang-Mangrove Garden in Hainan Province	**14**		[32]
				**15**	normal splenocytes (111.7 ± 1.1 μmol L^−1^) ConA-Induced T-Cell Proliferation (21.6 ± 1.7 μmol L^−1^) LPS-Induced B-Cell Proliferation (78.5 ± 1.3 μmol L^−1^)	
				**16**		
				**17**	ConA-Induced T-Cell Proliferation (42.35 ± 2.49 μmol L^−1^) LPS-Induced B-Cell Proliferation (88.19 ± 2.59 μmol L^−1^)	[33]
				**18**	HepG2 (59.14 ± 15.79 μmol L^−1^) Hela (5.82 ± 0.82 μmol L^−1^)	
5	*Pestalotiopsis* sp. HQD-6	fresh, healthy leaf of Chinese mangrove plant *Rhizophora mucronata*	Dong Zhai Gang-Mangrove Garden on Hainan Island, China	**19**		[34]
				**20**	Hela (50.42 ± 0.07 μmol L^−1^)	
6	*Talaromyces flavus* TGGP35	the stem of the mangrove plant *Acanthus* *ilicifolius*	Dongzhai Port, Haikou, Hainan Province	**21**		[35]
				**22**		
				**23**		
				**24**		
				**25**		
				**26**	antioxidant activity (0.14 mmol L^−1^)	
				**27**		[36]
				**28**		
				**29**	Hela (62.23 ± 0.23 μmol L^−1^)	
				**30**		
				**31**	antioxidant activity (0.40 mmol L^−1^)	
				**32**	Hela (57.14 ± 0.15 μmol L^−1^)	
				**33**	anti-insect activity against newly hatched larvae of Helicoverpa armigera Hubner (50–200 µgmL^−1^)	
7	*Fusarium* sp. 2ST2	healthy leaves of *Kandelia candel*	the South China Sea, Dong Zhai Harbor Mangrove Nature Reserve Area, Hainan Province, China	**34**	A549 (8.7 μmol L^−1^)	[37]
				**35**	A550 (4.38 μmol L^−1^)	
				**36**		
				**37**		
				**38**		
				**39**		
				**40**		
				**41**		
8	*Aspergillus* *fumigatus* HQD24	the flower of the Chinese mangrove plant *Rhizophora mucronata*		**42**	decreased ACAT2 inhibitory activity (12.0 mmol L^−1^)	[38]
9	*Nigrospora* *camelliae-sinensis* S30	mangrove *Lumnitzera littorea*		**43**		[39]
				**44**		
10	*Penicillium crustosum* SCNU-F0006	*Acanthus ilicifolius* L. mangrove plant	the Yangjiang Mangrove Nature Reserve in Guangdong Province	**45**	RAW 264.7 cells (above 50 μmol L^−1^) anti-inflammatory activity (42.22 ± 2.26 μmol L^−1^) DPPH radical scavenging activity (180.2 μmol L^−1^) antimicrobial activities against *Bacillus subtilis*, *Penicillium italicum*, and *Pseudomonas aeruginosa*	[40]
11	*Diaporthe* sp. ZJHJYZ-1	a fresh leaf of the semi-mangrove plant *Hibiscus tiliaceus* L.	Zhanjiang Mangrove National Nature Reserve in Guangdong Province, China	**46**		[41]
				**47**		
12	*Penicillium sclerotiorum* ZJHJJ-18	the stems of the mangrove plant *Hibiscus tiliaceus*	the shore of the Zhanjiang Mangrove Nature Reserve in Guangdong Province, China	**48**	inhibit LPS-induced NO production in RAW264.7 macrophage cells	[42]
				**49**	inhibit LPS-induced NO production in RAW264.8 macrophage cells	
				**50**	inhibit LPS-induced NO production in RAW264.9 macrophage cells	
				**51**	inhibit LPS-induced NO production in RAW264.10 macrophage cells	
				**52**	inhibit LPS-induced NO production in RAW264.11 macrophage cells	
				**53**	inhibit LPS-induced NO production in RAW264.12 macrophage cells	
				**54**		
				**55**		
				**56**		
13	*Penicillium herquei* JX4	the mangrove *Ceriops* *tagal*	the South China Sea	**57**	anti-inflammatory activities	[43]
				**58**	anti-inflammatory activities	
				**59**	anti-inflammatory activities	
14	*Phomopsis* sp. QYM-13	healthy leaves of *Kandelia candel*	the South China Sea, Dongzhai Harbor Mangrove Nature Reserve Area, Hainan Province, China	**60**		[44]
				**61**	MDA-MB-435 (4.9–8.2 μmol L^−1^)	
				**62**		
				**63**		
				**64**		
				**65**		
				**66**		
				**67**		
				**68**		
				**69**		
				**70**		
				**71**		
15	*Daldinia eschscholtzii* KBJYZ-1	the root of *Pluchea indica* Less	Zhanjiang Mangrove National Nature Reserve in Guangdong Province, China	**72**	anti-inflammatory activities (19.3 μmol L^−1^)	[45]
				**73**		
				**74**		
				**75**		
				**76**	anti-inflammatory activities (12.9 μmol L^−1^)	
16	*Aspergillus sydowii* #2B	the leaves of the mangrove plant *Aricennia marina*		**77**	inhibit the production of nitric oxide (NO) in lipopolysaccharide (LPS)-induced RAW 246.7 cells (40.15 μmol L^−1^)	[46]
				**78**	VCaP (20.06 ± 2.01 μmol L^−1^)	
17	*Aspergillus* *fumigatus* SAI12	leaves of mangrove plant *Sonneratia apetala* Buch.-Ham.	Dongzhaigang National Nature Reserve in south China’s Hainan Province	**79**		[47]
				**80**		
18	*Aspergillus* sp. GXNU-A9	a leaf of mangrove *Acanthus ilicifolius* L.	Qinzhou City, China	**81**	moderate inhibitory activity against nitric oxide (NO) production anti-inflammatory activity	[48]
19	*Aspergillus* sp. GXNU-Y65	fresh fruit of the mangrove plant *Kandelia candel*	Beihai, China	**82**		[49]
				**83**		
				**84**	anti-nonalcoholic steatohepatitis activity	
				**85**		
				**86**		
				**87**		
				**88**		
				**89**		
				**90**		
20	*Aspergillus* sp. GXIMD00016	Fresh leaves of *Kandelia candel*	Beihai Golden Bay Mangrove Reserve	**91**		[50]
21	*Aspergillus* QG1a	the mangrove *Kandelia candel*	the seaside of Qinzhou, Guangxi Province, China	**92**	A549 (59.283 μmol L^−1^) A2780 (46.197 μmol L^−1^) MIA PACA-2 (42.664 μmol L^−1^)	[51]
				**93**	A2780 (6.808 μmol L^−1^) MIA PACA-2 (15.400 μmol L^−1^)	
22	*Aspergillus* sp. GXNU-4QQY1a	healthy leaves of *Acanthus ilicifolius* L.		**94**	insecticidal activity against citrus psyllids (lethality values of 92.31 ± 6.20%)	[52]
				**95**	exhibited good insecticidal activity against citrus psyllids (lethality values of 87.80 ± 9.32%)	
23	*Didymella* sp. CYSK-4	a fresh branch of the mangrove plant *Pluchea* *indica*	Shankou Mangrove Na ture Reserve in Guangxi Province, China	**96**	A549 (11.0 μmol L^−1^)	[53]
				**97**	A549 (2.8 μmol L^−1^) KYSE 150 (5.9 μmol L^−1^)	
				**98**		
				**99**		
24	*Fusarium. proliferatum* NSD-1	a fresh twig from mangrove plant *Kandelia candel*	a Mangrove of Nansha District of Guangzhou in Guangdong Province, China	**100**	A549 (75.9 ± 10.4 μmol L^−1^) SW480 (37.5 ± 8.0 μmol L^−1^)	[54]
				**101**		
				**102**		
				**103**	moderate IL-1β inhibitory activity	
				**104**	A549 (24.9 ± 10.1 μmol L^−1^) SW480 (77.7 ± 3.6 μmol L^−1^)	
25	*Penicillium verruculosum* TGM14	mangrove *Xylocarpus granatum* Koenig	the South China Sea	**105**		[55]
26	*Penicillium* sp. GXIMD 03001	the rhizophoraceous mangrove *Kandelia candel*	the Beibu Gulf	**106**		[56]
				**107**		
27	*Amorosia* sp. SCSIO 41026	the leaf of *Avicennia marina* (Forsk.) Vierh.	the mangrove wetland in Zhanjiang, Guangdong province, China	**108**	inhibit LPS-induced NO production in RAW264.7 macrophage cells	[57]
				**109**		
				**110**		
				**111**	inhibit LPS-induced NO production in RAW264.7 macrophage cells	
				**112**		
